# Advancements and Future Prospects of CRISPR-Cas-Based Population Replacement Strategies in Insect Pest Management

**DOI:** 10.3390/insects15090653

**Published:** 2024-08-30

**Authors:** Yu Zhao, Longfeng Li, Liangzi Wei, Yifan Wang, Zhilin Han

**Affiliations:** College of Life Science and Technology, Gansu Agricultural University, Lanzhou 730070, China; fenger4499@163.com (L.L.); liangzi6898@163.com (L.W.); wyifan202408@163.com (Y.W.); lhan08380@gmail.com (Z.H.)

**Keywords:** population replacement, genetically edited insect, gene drive, CRISPR-Cas, insect pest management

## Abstract

**Simple Summary:**

Many insects are categorized as agricultural pests due to their ability to transmit diseases and damage crops, which results in significant economic losses. Scientists have proposed two main pest control strategies: population suppression, aimed at reducing the size or distribution of pest populations, and population replacement, which involves introducing genetically modified populations to replace wild pests after an initial release. Typically, population replacement strategies use gene drive systems to spread beneficial traits throughout the target population. Current promising gene drive systems include homing endonuclease genes (HEGs), *Wolbachia*, maternal-effect dominant embryonic arrest (Medea), and newly adapted CRISPR/Cas genome editing systems. This review provides an overview of the recent advancements in population replacement, including insights into the development, testing, and safe implementation of CRISPR-Cas-based gene drive techniques from laboratory settings to field applications. It also discusses recent developments, identifies research gaps, and offers a comprehensive analysis of genetic control strategies for insect pests.

**Abstract:**

Population replacement refers to the process by which a wild-type population of insect pests is replaced by a population possessing modified traits or abilities. Effective population replacement necessitates a gene drive system capable of spreading desired genes within natural populations, operating under principles akin to super-Mendelian inheritance. Consequently, releasing a small number of genetically edited insects could potentially achieve population control objectives. Currently, several gene drive approaches are under exploration, including the newly adapted CRISPR-Cas genome editing system. Multiple studies are investigating methods to engineer pests that are incapable of causing crop damage or transmitting vector-borne diseases, with several notable successful examples documented. This review summarizes the recent advancements of the CRISPR-Cas system in the realm of population replacement and provides insights into research methodologies, testing protocols, and implementation strategies for gene drive techniques. The review also discusses emerging trends and prospects for establishing genetic tools in pest management.

## 1. Introduction

Insects comprise the most numerous animal group, with over one million described species, representing more than half of all known living creatures on Earth [1]. The estimated total number of extant insect species equates to several million. Many insects are considered agricultural pests due to their ability to spread disease and damage crops, leading to substantial global economic losses annually [2,3]. Over recent decades, scientists have proposed various pest control methods using genetic engineering, primarily categorized into two strategies: population suppression and population replacement. Population suppression aims to decrease insect numbers or their geographical spread [4,5,6,7]. In contrast, population replacement strategies involve replacing wild-type pest populations with genetically modified strains or species, resulting in the genetically modified organisms occupying the ecological niche [8,9,10,11,12].

Suppression strategies include chemical insecticides, the sterile insect technique (SIT), the incompatible insect technique (IIT), the release of insects carrying a dominant lethal gene (RIDL), and biological control. These population suppression strategies have been extensively employed against various agricultural pest insects for decades. For instance, chemical insecticides have been widely utilized globally to control a variety of insect pests, offering advantages such as reduced labor and time requirements, low labor intensity, and high operational efficiency [13]. The sterile insect technique (SIT) is an environmentally friendly, non-resistant, non-polluting, and species-specific method used for the area-wide control of agricultural pests. It has demonstrated successful application in pests such as medflies, screwworms, fruit flies, moths, pink bollworms, codling moths, and tsetse flies [14]. The release of insects carrying a dominant lethal gene (RIDL), an advanced form of SIT, is particularly effective due to its fitness and cost advantages. RIDL has been successfully developed and tested in laboratory and open-field environments against pests like the diamondback moth, mosquito, and medfly [15]. The incompatible insect technique (IIT), akin to SIT and RIDL, relies on the sustained release of *Wolbachia*-induced cytoplasmic incompatibility (CI) to sterilize pests. Developed over 40 years ago, IIT has proven effective in controlling the vector-borne diseases of mosquitoes and medflies [16,17].

The population suppression methods using SIT, IIT, and RIDL techniques generally depend on long-term rearing and large-scale releases, which can be expensive to implement. These methods often encounter challenges such as inconsistent infrastructure availability, bottlenecks in efficient sex-sorting, quality control issues, extendibility problems, and suboptimal field performance. Furthermore, the repeated use of chemical insecticides can lead to the development of highly resistant insects with reduced susceptibility, contributing to the environmental pollution which is harmful to ecosystems and human health [18,19]. These factors collectively limit the effectiveness of these approaches [20]. Furthermore, most non-selective methods struggle to effectively target and control omnivorous pest insects with diverse host ranges, larvae that feed inside host fruits, and pests exhibiting opportunistic feeding and resting behaviors [19].

Therefore, there is a need to develop new pest control methods that can address these shortcomings. One promising strategy which is gaining attention is population replacement, where genetically modified populations replace wild pest populations following an initial ‘seeding’ release [21]. Population replacement strategies generally involve the deployment of gene drive systems, which combine a non-pestilent trait with a selfish genetic element that spreads rapidly within the target population once a threshold frequency is reached [22]. Compared to most conventional population suppression methods that rely on inundative releases and Mendelian inheritance patterns, gene drive strategies typically require fewer releases of individuals (either male or female) into the wild population. This makes them potentially more efficient and sustainable for long-term pest control solutions.

In this review, we consolidate the literature from the last 10 years regarding population replacement strategies for controlling agricultural insect pests. Our aim is to facilitate an understanding of the design, testing, and safe implementation of this approach from the laboratory setting to the field application. Additionally, we highlight advancements and identify areas requiring further investigation within population replacement research, thereby offering an overview of emerging insect pest management methodologies. Looking ahead, interdisciplinary collaboration including mathematical modeling, population surveys, legislative guidelines, ecology, and genetics will be essential for advancing this field of research.

## 2. Population Replacement Using Gene Drive Systems

### 2.1. Molecular Genetic Manipulation of Insects

**Figure 1:**
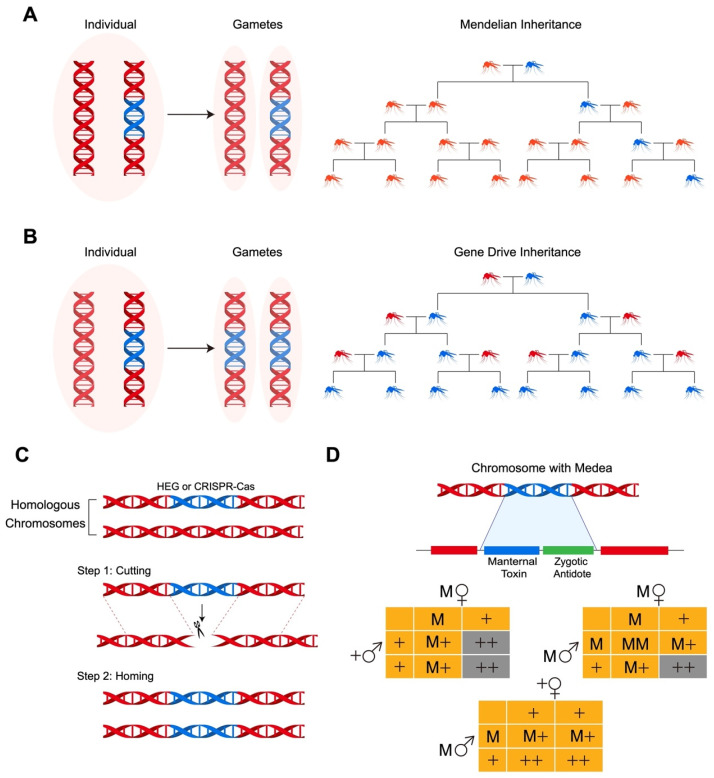
Gene drives manipulate inheritance to rapidly spread altered genes through populations. In Mendelian inheritance (**A**), offspring have a 50% chance of inheriting an altered gene (shown in blue), when an individual carrying the altered gene on only one of two homologous chromosomes is crossed with a wild-type (WT) individual (shown in red). Under gene drive inheritance (**B**), crossing a trans-heterozygous organism with a WT organism will result in the gene drive system converting WT chromosomes to the altered gene in the germline cells, if the process is confined to the germline. As a result, offspring will inherit the altered gene 100% of the time. This leads to the rapid replacement of WT organisms with a specific genotype over fewer generations. The spread of a selfish gene through a population depends on the gene drive system, the organisms, and the ecological conditions. In a diploid organism (**C**) with a site-specific HEG (homing endonuclease gene) or CRISPR/Cas (shown in blue) integrated into one homologous chromosome, the endonuclease causes a DNA break in the wild-type chromosome. Homology-dependent repair then copies the modified allele into the wild-type chromosome. This process, called homing, results in HEG- or CRISPR-Cas-bearing gametes. The Medea (maternal effect dominant embryonic arrest) system (**D**) uses a maternal toxin and a zygotic antidote to drive gene spread. Heterozygous Medea females crossed with WT or heterozygous Medea males produce only viable embryos that inherit the Medea element. When Medea-bearing males are crossed with WT females, all embryos survive because the maternal toxin is not expressed. This system creates a frequency-dependent fitness advantage by killing the offspring of Medea heterozygous mothers that did not inherit Medea, allowing the spread of a cargo gene. The CRISPR-Cas-based toxin-antidote system uses a maternal toxin and zygotic antidote to promote the survival of offspring with the toxin-antidote element, thereby spreading a cargo gene throughout the population. Lethality is indicated by a gray background, +: WT gamete; M: Medea-bearing gamete; M♀: heterozygous Medea female; M♂: heterozygous Medea male; +♀: WT female; +♂: WT male.

To achieve effective population replacement for insect pest control, a gene drive system capable of biasing inheritance in its favor is essential. This requires the introduction of a selfish genetic element that autonomously spreads with super-Mendelian inheritance (greater than a 50% likelihood) within the target population, ultimately leading to the replacement of the local population with a specific genotype. This introduced construct could potentially disrupt or introduce a desired gene that lacks disease transmission capability or exhibits reduced virulence and damaging characteristics. Several gene drive approaches are currently under investigation, including HEGs (homing endonuclease genes) [23], Medea (maternal effect dominant embryonic arrest) [24,25], and CRISPR-Cas (clustered regularly interspaced short palindromic repeats-CRISPR-associated proteins) systems (Figure 1). While the concept of using gene drives to manage insect pests is not new, achieving this goal remains challenging, hampered by scientific and technical obstacles. Recent advancements in the CRISPR-Cas system, a precise site-specific gene editing technology, offer promising solutions to overcome some of these challenges. Therefore, we primarily focus on the application of CRISPR-Cas technology in gene drive systems.

The CRISPR-Cas system is a genetic engineering tool derived from an adaptive immune defense mechanism found in many archaea and bacteria. It integrates partial sequences of foreign genomes between the CRISPR repeat sequences, which are organized as arrays within specific genomic loci. These sequences are transcribed, producing RNAs that guide Cas proteins to the target and cleave the genomes of phages or plasmids. Among the various Cas proteins, several form a cascade complex which is crucial for the processes at the CRISPR loci and for DNA cleavage. There are three primary types (I, II, and III) of CRISPR-Cas systems, each characterized by distinct sets of Cas genes [26]. Type II CRISPR systems utilize the RNA-guided endonuclease *Sp*Cas9 (formerly Csn1) from *Streptococcus pyogenes* to cleave DNA [27,28]. In this two-component system, protospacer sequences derived from invading DNA are processed into CRISPR RNAs (crRNAs), which then hybridize with a transactivating CRISPR RNA (tracrRNA) to form a complex, guiding Cas9 to cleave the complementary target DNA sequence. Researchers have simplified this system by engineering the tracrRNA duplex into a programmable chimeric single-guide RNA (sgRNA) [29,30]. By altering the first 20 nucleotides of the sgRNA sequence, different DNA sequences can be targeted, enabling multiplexed target recognition. Additionally, the versatility of the CRISPR-Cas9 system, achieved through component modifications, facilitates gene repression, activation, epigenome editing, and RNA editing capabilities [31].

The CRISPR-Cas system offers an ideal tool for constructing gene drives. This system functions by transferring drive alleles from one chromosome to another at a specified locus that matches the sgRNA-guided genome sequence. In germ cells, this method converts heterozygotes to homozygotes, facilitating super-Mendelian inheritance. Engineered for activity in the germline or embryo of diploid organisms, gene drives ensure the production of CRISPR-Cas-bearing gametes, with heterozygous parents primarily transmitting the gene drive. This mechanism exceeds the typical 50% inheritance rate expected for a single copy of the drive allele. In principle, CRISPR-Cas-mediated gene drives could be utilized to propagate specific genes throughout populations of engineered organisms by releasing a small number of gene drive heterozygotes, potentially replacing wild populations within a few generations [32].

### 2.2. Performance of CRISPR-Cas-Mediated Gene Drive Populations

The CRISPR-Cas-mediated gene drive in insects was first reported in *Drosophila melanogaster* in March 2015 [33]. Subsequent experiments successfully applied this system to *Anopheles gambiae*, *Anopheles stephensi*, *Aedes aegypti*, *Drosophila suzukii, Ceratitis capitata*, and *Plutella xylostella* [34,35,36,37,38,39,40,41].

Cas9 was utilized to integrate trait or cargo genes, with Gantz pioneering the mutagenic chain reaction (MCR) in *D. melanogaster*. The MCR achieved transmission efficiencies averaging 97%, significantly exceeding Mendelian inheritance expectations for a recessive X-linked yellow locus [33]. Oberhofer et al. developed the ClvR (Cleave and Rescue) system in *D. melanogaster*, a toxin-antidote gene drive on an autosomal chromosome targeting an essential X-linked gene [37]. It imposes fitness costs on wild-type offspring, favoring ClvR-bearing chromosomes through gene conversion and maternally transmitted Cas9 activity. ClvR^tko^ effectively deactivated over 99% of the targeted genes with minimal functional resistance mutations. Another ClvR-based strategy developed by Oberhofer et al. involves placing two distinct ClvR elements that target different essential genes at the same genomic locus but on different homologous chromosomes. The second-generation element incorporates a rescue mechanism for the existing first-generation element within the population. Upon introduction into a population already carrying the first-generation element, the second-generation element spreads to fixation, displacing the latter [42]. ClvR has shown effectiveness across multiple highly conserved genes, suggesting its adaptability and potential for implementation in target species. Furthermore, the ClvR design enables the use of multiple gRNAs as toxins, thereby reducing the likelihood of developing resistant alleles that restore target gene function.

Champer et al. also created a “same-site” toxin-antidote drive in *D. melanogaster*, combining both elements within a recessive lethal gene to potentially enhance efficacy using the native promoter [43]. The study demonstrates that the TARE (CRISPR Toxin-Antidote Recessive Embryo) system effectively biases inheritance of the drive allele in a large *Drosophila* population cage. Within six generations of a modest release, the drive allele spread to all individuals without any resistance allele formation observed. A split gene drive in *D. melanogaster* targeting the haplolethal gene RpL35A used two guide RNAs and a gRNA-resistant rescue allele [44]. By generation six, the drive allele reached fixation from an initial frequency of 32% when mixed with Cas9 homozygous eggs. Efficient homing and fixation were achieved with Cas9 expression from a separate locus. Offspring from crosses between heterozygous parents and the wild-type demonstrated high inheritance rates, but progeny from gene drive heterozygous females and wild-type males had reduced survival, likely due to maternal Cas9 effects. All these gene drive systems were designed to target a vital gene for inactivation, which was subsequently rescued by inserting a recoded transgene at a different locus in the genome.

Incorporating functional recoded versions of genes into drives in modification systems has been demonstrated to significantly improve their performance [45]. Nash et al. investigated the minimal genetic elements necessary for autonomous gene drives in *D. melanogaster* by integrating a Cas9 open reading frame with an intronic gRNA into the *rcd-1r* locus, demonstrating efficient propagation in both male and female germlines [46]. The study highlighted the potential applications in modular drive and effector functions, alongside considerations of the biosafety implications for minimal and potentially recoded gene drives. Sanz Juste et al. first introduced a Cas12a-driven gene drive system regulated by temperature [47]. Higher temperatures increased the super-Mendelian inheritance rates for both alleles (*e1*-GD and *e4*-GD). At 18 °C, *e4*-GD showed no gene drive activity, suggesting it can be temperature-controlled. These findings pave the way for advancing Cas12a-based gene drive systems in insects, aiming to improve population control strategies across different pests.

By late 2015, Gantz et al. adapted the CRISPR-Cas-based gene drive to *A. stephensi*, achieving transmission rates of over 99.5% with an engineered synthetic construct [34]. Hammond et al. applied a CRISPR^h^ gene drive in *An. gambiae*, disrupting three female fertility genes with high transmission rates [35]. The study proposed a new strategy to suppress malaria spread, despite reduced fertility in the offspring due to Cas9-induced somatic mutations. A 25-generation cage study showed an initial rise and subsequent decline in gene drive frequency due to resistant target site mutations. By the 25th generation, transgenic heterozygotes fell below 20%, revealing challenges in sustaining the gene drive efficiency over time. In *An. gambiae*, a CRISPR gene drive disrupted the *doublesex* (*dsx*) gene, which is crucial for sex determination. Homozygous females with the disrupted allele were sterile with intersex traits, while males developed normally. The gene drive achieved >95% transmission efficiency in the offspring of heterozygous parents and reached 100% frequency within 12 generations in cage studies starting at 12.5%. Minimal indels (1.16%) at the gRNA target site suggest low resistance potential [36]. An efficient gene drive strain, AgNosCd-1, has been successfully demonstrated in *An*. *gambiae*. Integrated into the *Agcd* gene, it incorporates fluorescent and morphological markers to track its spread. Initial outcrossing with wild types resulted in >95% drive propagation in the offspring, maintaining an average efficiency of 96.7% over four generations. The gene drive imposes a low fitness penalty in both heterozygous and homozygous states, highlighting its potential as a platform for introducing desired cargo genes into populations [48]. The study documents resistance developing over generations in response to a synthetic gene drive, emphasizing the need to address resistance in gene drive design. The AgNosCd-1 gene drive system is expected to be among those prioritized for evaluation in the upcoming phase of open field trials.

Adolfi developed a recoded gene drive rescue system for modifying *Anopheles stephensi* populations by inserting a gene drive with recoded cDNA sequences into a critical locus, ensuring viability and fertility in a homozygous state [49]. This approach eliminates non-functional NHEJ alleles through dominant lethal or sterile effects, as demonstrated at the *kh* locus. Cage trials showed that a single release of gene drive males achieves an effective population modification, with ≥95% of mosquitoes harboring the drive within 5–11 generations across different initial release ratios. Hammond et al. tested new constructs targeting a female fertility locus prone to resistance, aiming to quantify reduced resistance mutations by enhancing female fertility and reducing embryonic end-joining [50]. They investigated the spread of the *zpg*-CRISPR^h^ gene drive in mosquito populations, observing rapid propagation to over 97% frequency in four trials spanning 4–10 generations. The gene drive sustained frequencies above 95% for at least three generations before encountering resistance. The initial release frequency (10% or 50%) did not significantly influence its spread, demonstrating effective invasion dynamics compared to the first-generation gene drive (*vas2-CRISPR^h^*).

In other insects, Xu et al. developed a CRISPR-Cas-based split drive system in *Plutella xylostella*, but it failed to produce homing-based progeny [41]. Asad et al. created a CRISPR-Cas gene drive construct for the same pest, incorporating Cas9, an EGFP marker gene, and a gRNA targeting *Pxyellow*. Their work achieved HDR efficiencies of 6.67–12.59% and generated resistant alleles through NHEJ at rates of 80.93–86.77%. Male-derived progeny showed higher gene drive efficiency than females, indicating potential trait inheritance [51]. In contrast, Meccariello characterized the first gene drives in *Ceratitis capitata* and developed a gene drive targeting the *transformer* gene using the *vasa* promoter. This gene drive system demonstrated an 83.1% transmission rate, indicating that the medfly is highly suitable for homing-based gene drive strategies [40].

Table 1 summarizes homing efficiencies across various generations of CRISPR-Cas-mediated gene drive systems. These studies highlight considerable variability in gene drive efficiencies, ranging from approximately 37% to 99% in *D. melanogaster* and from 0 to 99% in mosquitoes. This variability highlights the influence of factors such as the timing and level of Cas expression, organism-specific traits, various gene drive modular elements and their positions, as well as the specific genomic targets.

## 3. Ideal Traits for Gene Drives in Pest Control

CRISPR-Cas-mediated gene drive systems offer substantial potential for pest control by replacing harmful wild-type alleles with gene drive alleles. This approach allows the engineered individuals to replace the target populations rapidly, even with a low initial frequency. Successful implementation of this strategy as a population replacement tool for pest management requires the selection of key phenotype-related genes for targeting or insertion [62,63]. Potential applications include reducing fertility, limiting development, altering food consumption, impacting mating competitiveness, skewing sex ratios, curtailing migration, decreasing longevity, reducing stress resistance, modifying behavior, affecting immune function, and lowering disease transmission—all aimed at diminishing fitness traits or species’ pest potential.

The high efficiency of the CRISPR-Cas-mediated gene drive ensures that genetically modified organisms can spread readily, even when their insertions have fitness costs [50]. Alternatively, CRISPR-Cas-based population replacement strategies can lead to population suppression by imposing significant fitness costs (e.g., sterility) or gender biases (e.g., driving Y-chromosomes, X-chromosome shredders) [64,65,66]. Moreover, these systems can propagate recessive mutations causing lethality, infertility, or single-sex offspring [67]. Theoretically, releasing a small number of insects carrying such gene drive alleles can lead to the accumulation of heterozygous individuals producing predominantly gene drive gametes. Over multiple generations, mating among these heterozygotes could potentially suppress or collapse the population, contingent upon driving efficiency and fitness costs. Laboratory studies have demonstrated this strategy using HEGs and the CRISPR-Cas system [36,68].

### 3.1. Blocking Pathogen Transmission

Vector insects frequently inflict severe damage on the agricultural sector and pose health risks by transmitting pathogens such as viruses, parasites, and bacteria to humans or other arthropods. Typically, these vectors acquire infectious agents from infected hosts and subsequently transmit them to new hosts through multiple feeding cycles interspersed with egg-laying phases. A functional CRISPR-Cas-mediated gene drive represents a highly efficient strategy for controlling insect vectors. This approach involves replacing disease-carrying native strains with disease-resistant variants [69]. The primary objective of such gene drives is to reduce vector competence by integrating anti-pathogen genes into the driving alleles. These anti-pathogen genes may encompass exogenous peptides targeting pathogens, single-chain antibodies (scFvs), and regulators associated with immune functions. For instance, in mosquitoes, the elevated expression of m2A10 scFvs targeting the *P. falciparum* circumsporozoite protein inhibits sporozoites from invading salivary glands [70]. Incorporating either m4B7 or m1C3 scFv transgenes, or employing versatile transgenic multistage effector genes as demonstrated in one recent study, leads to a nearly complete inhibition of parasite transmission [71].

### 3.2. Manipulation of Sex Ratios

In many pest insects, population size primarily hinges on the reproductive capacity of the females. Female insects, acting as vectors, transmit numerous insect-borne pathogens such as plasmodium, tospovirus, and Puccinia. Segregation-distorting gene drives are selfish genetic elements that manipulate the process of gametogenesis. Therefore, the implementation of gene drive systems has the potential to skew population sex ratios away from the typical Fisherian ratio of 1:1 male to female, favoring a male-dominated population by promoting ‘maleness’ or altering reproductive dynamics [72]. Persistent male bias over successive generations can precipitate rapid population decline, with complete eradication possible when populations become exclusively male [73]. For instance, the gene drive construct dsxF^CRISPRh^, targeting the *doublesex* gene to induce sterility in homozygous females, caused the complete collapse of a confined population of 600 *An. gambiae* mosquitoes within 12 generations, with dsxF^CRISPRh^ heterozygotes initiated at a 12.5% allelic frequency [36]. Similarly, the SDGD^dsx^ gene drive construct, employing *zpg*-Cas9, U6-gRNA, and *beta2^244^*-I-PpoI to target the *doublesex* gene and ribosomal DNA repeats of the X chromosome, demonstrated a pronounced male-biased sex ratio distortion and achieved population elimination within 13 generations starting from a 10% release of SDGD^dsx^-heterozygous males [61]. This approach of genetic sex-sorting offers more effective population suppression compared to methods like the SIT. Identification of sex determination genes like *doublesex* and *transformer* presents opportunities to manipulate population sex ratios, potentially offering a powerful strategy for controlling invasive pests, particularly vector species, through CRISPR-Cas-mediated gene drives.

### 3.3. Manipulation of Feeding Behaviors

Insects exhibit behavioral responses through sensory signals integrated within the central nervous system. These signals arise from physical and chemical stimuli detected by exteroceptors (sensing external stimuli), enteroceptors (sensing internal stimuli), and proprioceptors (sensing body position) [74,75,76]. Receptor cells convert these sensory inputs into electrical signals, which are distributed across various systems, including vision, hearing, smell, taste, and touch [68]. Olfactory cues, primarily processed by olfactory sensory neurons (OSNs), play a crucial role in insect behavior, guiding responses to pheromones, food, predators, shelter, and conspecifics [77,78]. Molecular studies on insects such as flies, moths, bees, and mosquitoes elucidate how olfactory systems regulate behaviors related to age, feeding, circadian rhythms, and mating [79,80,81]. Similarly, the gustatory system enables insects to detect non-volatile phytochemicals through taste sensilla strategically located on the external and internal tissues, aiding in decisions related to feeding, mate selection, and oviposition [82,83,84,85]. Gustatory receptors (GRs), analogous to olfactory receptors (ORs), are involved in chemosensation, encompassing the responses to pheromones and somatosensory stimuli [86,87,88].

**Figure 2:**
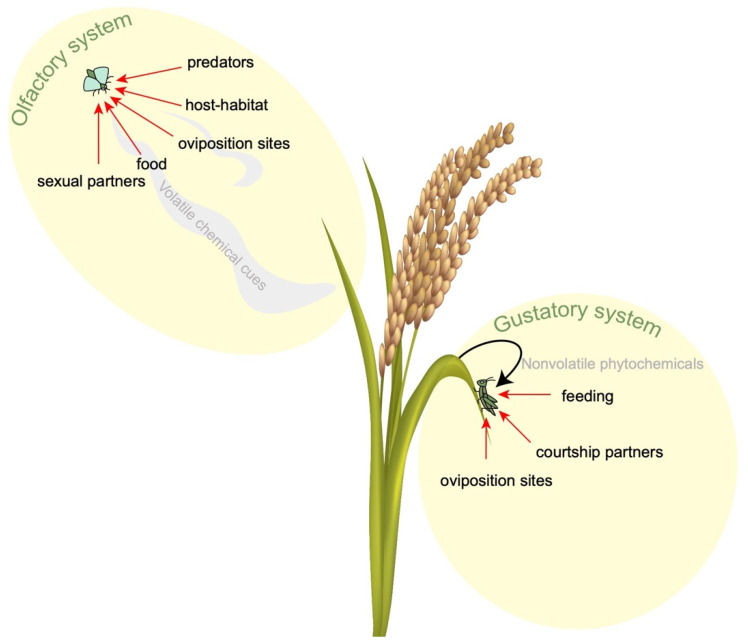
The concept involves utilizing a gene drive to manipulate insect pest behavior by targeting their sensory systems. Insects utilize their olfactory system to detect volatile pheromones and environmental odors, influencing behaviors such as food source localization, host selection, mating, oviposition, and predator avoidance. Their gustatory system plays a role in detecting nonvolatile phytochemicals, which impacts feeding, mating, and oviposition decisions. The application of a gene drive to manipulate these sensory mechanisms represents a promising avenue for sustainable insect pest management strategies.

CRISPR-Cas-mediated gene drive systems offer a promising approach to manipulating pest behaviors—such as feeding, food location, and mating—by targeting the sensory systems. This technique holds potential for sustainable insect pest management and disease control while concurrently safeguarding crops. For example, manipulating food-finding behavior, which is critical for the plant-feeding pests attracted to pathogen-infected plants, may reduce disease spread [89,90,91,92]. Plant viruses have the ability to influence the behavior of insect vectors both directly and indirectly by modifying plant biochemistry. Frequently, these behavioral changes result in a higher rate of interactions between the host plants and the vectors, thereby facilitating greater virus spread within the ecosystem. Additionally, gene drive systems targeting vector sensory responses to pathogen-induced chemicals could provide a sustainable strategy for insect-vector management (Figure 2). Enhancing natural enemies of insect pests through genetic strategies also presents economic benefits due to the precision of field applications.

### 3.4. Manipulation of Migration

Flight-mediated migration is a crucial adaptive behavior among insect pests, enabling them to exploit new environments in response to environmental variations [93]. Most insects exhibit migratory polymorphism, controlled by both endogenous genetic circannual rhythms and external factors [94,95]. The wing dimorphism of insect vectors is a crucial factor for the long-distance spread of pathogens and the development of widespread epidemics. Extreme cases demonstrate distinct morphological adaptations: migratory individuals develop long-winged forms, while flightless individuals evolve short-winged or wingless morphs with non-functional flight muscles, limiting their dispersal capabilities [96,97]. Research on wing dimorphism mechanisms in holometabolous insects (e.g., Diptera, Coleoptera, Lepidoptera) has identified a single genetic locus controlling this trait [98,99], whereas in hemimetabolous insects (e.g., Orthoptera, Hemiptera), the mechanism involves multiple gene interactions [100,101]. For instance, the rice stripe virus (RSV), transmitted by planthoppers, induces the development of long-winged males. The proportion of long-winged males correlates with RSV infection, independent of viral titers. The planthopper-specific gene *Encounter*, highly expressed in the testes, is crucial for this morph. *Encounter* is upregulated during early larval stages and influences male wing dimorphism via the insulin/insulin-like growth factor pathway, downstream of *Akt*, which is activated by RSV infection. The study demonstrates that a plant virus directly regulates wing dimorphism in its insect vectors, suggesting a method to disrupt viral spread [102].

Insects migrating longer distances to their overwintering sites generally exhibit longer and larger wings, suggesting that wing length and area are subject to positive selective pressure during migration [103]. Therefore, migration manipulation is not restricted to winged insects with dimorphism; insects without wing dimorphism might also be targeted. For example, migration behavior can be manipulated by targeting the genes related to long-distance migration (e.g., wing development, flight) in a pest using a gene drive. Given that thermal gliding is a key flight strategy for long-distance migration in insects, a reduction in wing area can severely impair their survival. Damaged wings lead to a progressive shift from gliding to flapping flight, resulting in increased energetic costs [104].

Strategies aimed at population replacement may offer greater resilience against pest population migrations into and out of release areas. Specifically, manipulating migration behavior through gene drive technologies which target the genes associated with long-distance flight in pests can potentially fix genetically modified alleles in the released population. This approach not only prevents reinvasion by wild pest populations capable of long-distance dispersal but also restricts the migration of pests out of the release area.

## 4. How Is a CRISPR-Cas-Mediated Gene Drive Made and Introduced?

**Figure 3:**
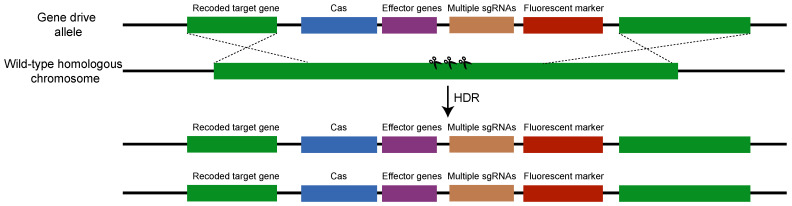
Schematic depiction of a non-split CRISPR-Cas-mediated gene drive mechanism. The construct comprises essential components: a Cas gene, sgRNA, effector genes, and flanking homology arms (illustrated in green). A fluorescent marker gene facilitates the identification of transgenic progeny. Upon insertion into a chromosome, independent expression of Cas and sgRNAs initiates a DSB on the homologous chromosome within germ cells. The inserted gene drive cassette remains intact due to disruption at the sgRNA recognition site. Repair via HDR results in duplication of the entire cassette into the wild-type homologous chromosome, leading to the presence of two gene drive cassettes in the genome, predominantly in sperm or eggs. Split gene drives, separating sgRNA from an unlinked Cas cassette to induce biallelic mutations, exhibit efficiency comparable to non-split constructs, maintaining the foundational gene drive principle.

The CRISPR-Cas-mediated gene drive construct typically comprises four elements: a codon-optimized Cas gene, an sgRNA gene with a specific target site, an effector gene conferring the desired phenotype, and homology arms flanking the Cas-sgRNA cassette to enable insertion at the target site [105]. A fluorescent marker gene may be included for easier identification of the transgenic progeny. Upon insertion of one copy of the gene drive cassette into a chromosome, Cas and sgRNA are independently expressed, causing a double-strand break (DSB) on the homologous chromosome lacking the gene drive [106]. This results in the gene drive cassette being copied into the wild-type homologous chromosome via homology-directed repair (HDR), producing two copies of the gene drive in the genome (Figure 3). The effectiveness of CRISPR-Cas-mediated gene drive systems for population replacement in controlling insect pests relies on several key factors: the selection of suitable promoters that specifically activate during germline cell development and gametogenesis, the phenotypic consequences of gene disruptions or insertions, the genetic stability of gene drive constructs throughout propagation, the associated fitness costs, and the efficiency of genetic transmission across generations. The application of gene drive systems to agricultural pests remains in the preliminary stages compared to their implementation in flies and mosquitoes, primarily due to these complexities.

Significantly, a germline-specific promoter employed to drive Cas expression can facilitate high rates of HDR-mediated homing exclusively within germ-line precursor cells destined to differentiate into sperm or eggs. Cas is under the control of various housekeeping and tissue-specific promoters used in loss-of-function studies across multiple species [107,108]. The majority of CRISPR-Cas gene drive constructs feature Cas expression driven by the *vasa* ortholog promoter, initially characterized in *D. melanogaster*, where it is active in both male and female germlines [109]. However, *vasa*’s expression extends beyond the germline, potentially leading to frequent non-homologous end-joining (NHEJ) events and the emergence of drive-resistant loci following DSBs. An improved strategy involves utilizing the *nanos* promoter to restrict Cas expression to male and female germ cells, which has shown reduced somatic leakage and embryonic toxicity compared to *vasa* [110]. Nonetheless, resistance alleles can still emerge pre- and post-fertilization with *nanos*-Cas gene drive constructs due to the maternal deposition of Cas-induced NHEJ alleles in genetically diverse populations [52]. Thus far, several germline-specific candidate promoters such as *beta2-tubulin*, zero population growth (*zpg*), *Rcd-1r*, and *Sry-α* have been identified in various gene drive constructs tested in confined insect populations, demonstrating minimal occurrence of NHEJ alleles with these promoters [36,54,61]. However, most of these promoters were discovered in model insects like *Drosophila* and mosquitoes, underscoring the urgent need for additional germline-specific promoters across different insects to optimize CRISPR/Cas-mediated gene drives in pest management. Additionally, codon optimization of the Cas nuclease is crucial. Codon usage bias varies widely between bacteria and insects, influencing Cas protein expression levels heterogeneously in the target organisms [111,112]. Codon-optimized Cas sequences have been shown to enhance expression in non-native hosts [113,114,115,116]. Moreover, the 5′-untranslated region (5’-UTR) and 3′-untranslated region (3′-UTR) play critical roles in regulating gene expression and homing efficiency [117,118]. For instance, the first intron of *αTub84B* could enhance Cas expression when inserted into the UTR of the Cas cassette [119,120]. Similarly, both the 5’-UTR and 3′-UTR of the β2-tubulin gene (encoded by *βTub85D*) have been demonstrated to improve homing efficiency in the male germline when positioned upstream and downstream of the Cas coding sequence [66,121].

Expressing sgRNA using an appropriate promoter is similarly a critical step in the CRISPR-Cas-mediated gene drive. Typically, sgRNA cassettes in gene drive constructs are driven by the RNA polymerase III-dependent U6 promoter. U6 small nuclear RNA (snRNA), a conserved component of the spliceosome found widely across species, serves this role and has been characterized in numerous organisms. The U6 promoter is well-suited for transcribing various small RNAs with precisely defined 5’ and 3’ ends, facilitating ubiquitous and high-level expression of sgRNAs [122,123]. Optimal transcription from the U6 promoter favors a guanine as the initiation site, necessitating sgRNA target sequences to start with 5’G-N19-NGG-3′ [124]. However, studies have demonstrated a tolerance for extra nucleotides mismatched at the 5′ end of sgRNA, allowing for flexibility in the target sequence selection by appending a guanine nucleotide prior to the target sequence, thereby conforming to 5’G-N20-NGG-3′ [125]. Alternatively, U3, H1, or RNA polymerase II promoters, including those identified in cell- or tissue-specific genes with variable transcription initiation sites, can be utilized for sgRNA expression to achieve precise spatio-temporal gene modification [126,127,128,129]. The variable region of the sgRNA provides a target site located on the homologous chromosome, enabling the placement of sgRNAs in tandem within gene drive constructs to simultaneously target multiple sites. To increase the likelihood of successful mutagenesis, it is essential to design and evaluate multiple sgRNAs targeting a specific gene before selecting the optimal one for use in the gene drive. The tRNA sequences could be inserted between multiple sgRNAs in the gene drive design due to their superior processing efficiency and the challenges posed by repetitive sequences during plasmid preparation [130].

The effector gene can be tightly linked with Cas and sgRNA genes that confer the desired phenotypes under the control of sex- or tissue-specific promoters, enabling expression in the appropriate pest tissues. The gene drive cassette, within the donor plasmid containing regions homologous to the target locus, is typically delivered to insects by microinjection into pre-blastoderm embryos. To enhance the germline transformation and insertion efficiency of the gene drive construct, additional dsRNA related to the NHEJ pathway (e.g., *Ku70*, *Ku80*) could be included in the injection [51,131]. The entire gene drive construct is copied into the homologous site by the cell for HDR, demonstrating the stable integration of at least ~17 kb of exogenous genes into the insect genome [34]. Importantly, transgenic progeny with non-visible phenotypes are challenging to identify and maintain; however, this can be facilitated by incorporating a fluorescent protein (e.g., EGFP, mCherry, DsRed) under the control of a strong promoter into the donor construct.

The CRISPR-based gene drive system requires further refinement before practical implementation. One critical concern is its evolutionary stability. While studies demonstrate an expected increase in the frequency of the gene drive locus over several generations, long-term effectiveness is limited by the emergence of resistance alleles in diverse natural populations, hindering the conversion of drive heterozygotes into drive homozygotes [132,133,134]. Resistance often arises from Cas-induced DSBs repaired via NHEJ or MMEJ rather than drive incorporation through HDR, particularly in mutated target sites resistant to further sgRNA recognition [133]. The rate of NHEJ-mediated repair varies widely among the species, target sites, and developmental stages during which DSBs occur [135,136]. Mathematical modeling suggests that the formation of such resistance alleles could severely impede the spread of gene drive constructs in wild populations, especially when they confer a fitness cost [137,138]. Additionally, pre-existing resistant alleles due to single-nucleotide polymorphisms (SNPs) may already exist in diverse wild populations before deployment. Enhancing the genetic stability of gene drive constructs has been proposed by using multiple sgRNAs closely spaced along the target site. In an effort to enhance stability, researchers modified the CRISPR-based *dsx* gene drive system by incorporating the X-chromosome shredding nuclease I-PpoI. This adaptation resulted in a male-biased sex ratio and ultimately caused the collapse of a small laboratory cage population [61].

Recent gene drive efforts have focused on creating homing split drives with a modified backup of the target gene to combat resistance. Adolfi et al. devised a recoded gene drive rescue system for *Anopheles stephensi* to combat the fitness costs linked to nonfunctional target sites and to prevent the emergence of resistance alleles [49]. This system exhibited promise in multi-generational laboratory populations. Another successful approach, Home and Rescue (HomeR), demonstrated strong transmission rates and limited generation of resistant alleles in fly populations, proving its effectiveness in cage studies [56]. Champer et al. introduced a homing drive in *D. melanogaster* targeting a haplolethal gene using two sgRNAs and a rescue allele, which significantly reduced resistance allele prevalence [53]. The drive demonstrates high efficiency, with 91% inheritance of the drive allele from heterozygotes, and successfully spreads in population cage experiments, underscoring its effectiveness for precise genetic modification. Terradas et al. offers insights into the design of the sGD (split gene drive) system to precisely manage spread and containment within native populations [45]. Strategies proposed include utilizing a linked Cas9 source for the controlled introduction and removal of the endonuclease, adjusting drive intensity through lethal or sterile mosaicism, and enhancing population-wide propagation by varying Cas9 activity levels. These adaptable systems have the potential to maintain allelic drives that affect the inheritance of advantageous traits or to distribute the desired cargo, such as supplementary anti-pathogenic molecules, in addition to the initial modifications [139]. These innovations aim to enhance gene drive effectiveness by integrating rescue alleles and specific targeting strategies [46]. Moreover, designing gene drives that disrupt the target loci with high sequence conservation suggests functional constraints that deter sequence changes likely to impair function [140]. Future improvements might focus on targeting essential genes and integrating an enhanced rescue mechanism to reduce fitness costs and boost drive efficiency.

While the CRISPR-Cas system has proven to be effective and robust, embryo microinjection for genetic modification remains severely limited due to its technical demands, time consumption, and labor intensiveness. Specifically, population replacement necessitates mass-reared transgenic insects, and this low-throughput manual injection method significantly restricts the application of gene drive technologies. There is a pressing need for the development of high-throughput genome engineering approaches to deliver gene drive components in insects, such as electroporation or paratransgenesis [141,142]. A new method called ReMOT Control has been developed for delivering CRISPR components into developing oocytes within the adult ovaries of different insect species [143,144,145]. This approach presents a viable alternative to traditional embryo microinjection methods. While effective for knock-out gene editing, its potential to deliver HDR templates for knock-in modifications is still under investigation. Overcoming these technical challenges is expected with further research efforts.

## 5. Benefits and Risks of Gene Drives

CRISPR-Cas-mediated gene drives for population replacement in pest control have significant biosafety implications. Modifying organisms with gene drives effectively creates new species, which can be highly invasive and spread through populations despite fitness costs. This broad dispersal may lead to unintended propagation into non-target ecosystems, causing ecological changes due to irreversible replacement of wild-type populations [146].

The practical implementation of gene drives requires robust safeguards and strategies in genetic design and confinement methods to mitigate the associated risks. Given the possibility of escape from laboratory or field trials, it is crucial to secure gene drives to prevent accidental spread to natural populations. Current strategies, mainly relying on physical confinement, may not adequately reduce the risk of escape due to human error. Since only a few escapees are needed to establish a viable drive [147,148], additional safety measures are necessary for experiments involving those drives capable of indefinite spread. One approach involves using species- or sub-population-specific sgRNA target sites in the gene drive construct to restrict the spread of the drive system to the target population [149,150]. Alternatively, with high-threshold gene drives such as underdominance, modular, or split systems, individuals containing a specifically conserved motif could be initially released to establish a pest population. Subsequently, a second gene drive population delivering the desired payload could replace the established population, contingent upon confirming that the implanted sequence does not spread to non-target populations. Once released, a gene drive cannot be recalled if Cas and sgRNAs are within the same gene cassette. Therefore, a split gene drive system, which separates the sgRNA from an unlinked Cas cassette to induce biallelic mutations, is a safer strategy. This method restricts sustained spread to the offspring of homozygous parents lacking the Cas gene, significantly reducing the risk of accidental release and non-drive inheritance patterns of the sgRNA cassette in the population [38,45,55,151]. Such split systems have demonstrated comparable efficiency to non-split constructs in *D. melanogaster*, *Plutella xylostella*, and *An. gambiae* [41,43,152,153,154]. Lastly, gene drive systems must be designed to avoid foreseeable risks that could harm target populations or disrupt ecosystems [154].

Ideally, a reversible mechanism should be incorporated into the gene drive construct to facilitate the removal of effector genes in cases where unexpected negative effects arise in the replaced populations. Efforts to control homing drives have been explored through various approaches. These include constructs that can overwrite existing drives in *D. melanogaster* [155], methods in *Anopheles gambiae* to mitigate suppression drives by reducing genetic load [156], and chemical inducible systems for regulating Cas expression and drive efficiency in flies [157]. D’Amato et al. first investigated the effectiveness of an AcrIIA4-based anti-drive against a gene drive in *Anopheles gambiae* populations. The experiments confirmed the anti-drive’s efficacy under diverse conditions, thereby supporting its potential for controlling gene drives in insects [158]. These methods aim to limit the spread of gene drives or revert organisms to their wild-type phenotype. Prior to considering field trials, extensive laboratory studies and population cage experiments are essential [159,160]. The efficacy of effector genes should be evaluated across diverse genetic backgrounds and environmental conditions, progressing from contained trials to controlled field trials, with open field releases of gene drive-carrying individuals reserved for the project’s final stages. While CRISPR-Cas-mediated gene drive research is advancing rapidly in laboratory settings, it remains in an early proof-of-concept phase. Continuous assessment and replication of gene drive-mediated replacements are crucial before transitioning from laboratory research to real-world applications that are effective, safe, and sustainable. Assessing the safety of gene drive technology involves a comprehensive approach including environmental risk assessment, laboratory and field trials, and risk mitigation strategies. Ethical considerations and stakeholder engagement are crucial, along with compliance with regulatory frameworks. Ongoing monitoring and adaptive management are essential for detecting and addressing unintended consequences. Scientific peer review and transparency further ensure responsible development and deployment. Ultimately, the successful implementation of large-scale releases hinges on scientific and technological advancements, regulatory approval, and public acceptance.

## 6. Conclusions

Genetic modification techniques are increasingly appealing as alternatives to nonselective methods. Genetically edited insects are deployed as control agents with heritable modifications that alter their traits. CRISPR-Cas-mediated gene drive strategies for pest control represent a more advanced approach compared to other genetic control methods, offering high species-specificity and cost-effectiveness. Self-sustaining population replacement strategies aim to reduce pest populations by leveraging rapid spread through initial releases. In contrast, self-limiting population suppression strategies focus on sustained large-scale releases to diminish pest numbers in targeted areas [161]. These genetic pest control strategies rely on successful mating between modified and wild individuals, yet mating barriers—such as postzygotic and prezygotic barriers—may hinder gene flow. Natural selection and genetic drift can further impede mating between genetically edited insects and wild populations. Highly effective gene drive systems, known for their invasive potential in wild populations, offer significant advantages in genetic insect pest control. With the rapid advancements in CRISPR-Cas technology widely applied across various organisms, this breakthrough tool holds promise for realizing effective gene drives. The potential for gene drives to contribute to sustainable and effective insect pest control appears promising.

## Figures and Tables

**Table 1:** Homing efficiencies of genetically edited populations with CRISPR-Cas-mediated gene drive systems.

Organism	Gene Drive Construct	Cas9 Cassette	gRNA Cassette	Marker	Target Gene	Phenotype	Homing Rate (G1)	Reference
*D. melanogaster*	MCR	*vasa*-Cas9	U6:3-gRNA	–	*yellow*	yellow body color	97%	[33]
	–	*nanos*-Cas9 *vasa*-Cas9	U6:3-gRNA	*3xP3-DsRed*	*yellow*	yellow body color	62% 52%	[52]
	–	*vasa*-Cas9	U6:3-gRNA	*3xP3-DsRed*	*yellow* *white*	yellow body color white-eye	37–53% 56%	[53]
		*nanos*-Cas9	U6:3-gRNA	*3xP3-DsRed*	*white* *white*-2gRNA *cinnabar* *cinnabar* *yellow*	white-eye white-eye brilliant orange eye brilliant orange eye yellow body color	59% 76% 54% 38% 40–62%	
	CHE	*Rcd*-*1r*-Cas9	U6:3-gRNA	*3xP3-DsRed*	*transformer*	sex conversion from females to males	56%	[54]
	ClvR	*nanos*-Cas9	U6:3-gRNA	*3xP3-GFP*	*tko*	lethality	99%	[37]
	tGD	*vasa*-Cas9	U6:3-gRNA	*3xP3-DsRed* *3xP3-EGFP*	*yellow* *ebony* *white*	yellow body color dark body color white-eye	67–98%	[55]
	ClvR	*nanos*-Cas9	U6:3-gRNA	*3xP3-GFP* *opie-td-tomato*	*dbe* *TfIIA-S*	recessive lethal	>99% in ♀; >94.7 to >99% in ♂	[42]
	TARE	*nanos*-Cas9	U6:3-gRNA	*3xP3-DsRed* *3xP3-GFP*	*hairy*	recessive lethal	88–95% in ♀	[43]
	–	*nanos*-Cas9	U6:3-gRNA	*3xP3-DsRed* *3xP3-GFP*	*RpL35A*	haplolethal	91%	[44]
	sGD	*nanos*-Cas9 *vasa*-Cas9	U6:3-gRNA	*3xP3-tdTomato* *3XP3-eGFP* *Opie2-DsRed*	*rab5* *rab11* *spo11* *prosalpha2*	recessive lethal recessive lethal sterility recessive lethal	64.8–99.9%	[45]
	HomeR	*nanos*-Cas9	U6:3-gRNA	*3xP3-EGFP*	*PolG2*	lethality	99.6% in ♀; 75.0% in ♂	[56]
	–	*nanos*-Cas9	U6:3-gRNA	*3xP3-DsRed*	*yellow-g*	recessive female sterility	86.4% in ♀; 90.4% in ♂	[57]
	–	*rcd*-*1r*-Cas9	U6:3-gRNA	*3xP3-CFP*	*rcd-1r*	male fertility	77.1% in ♂; 80.5% in ♀	[46]
	TARE TADE	*nanos*-Cas9	U6:3-gRNA	*3xP3-DsRed*	*RpL35A* *hairy*	haplolethal recessive lethal	51–54%	[58]
	–	*vasa*-*Cas12a*	U6:3-gRNA	*Opie2-GFP*	*ebony*	dark body color	52–89%	[47]
	–	*nanos*-Cas9 CG4415-Cas9 *rcd*-*1r*-Cas9	U6:3-gRNA	*3xP3-DsRed* *3xP3-GFP*	*doublesex*	dominant female sterility	70.0–85.5%	[59]
*Drosophila suzukii*	–	*nanos*-Cas9	U6:3-gRNA	*pUb-DsRed* *hsp83-ZsGreen*	*doublesex*	dominant female sterility	94–99%	[39]
	DsTdc2^CRISPR^	*vasa*-Cas9	U6:3-gRNA	*PUb-DsRed*	*Tyrosine decarboxylase 2*	recessive female fertility	53.6–58.2%	[60]
*Anopheles stephensi*	AsMCRkh2	*vasa*-Cas9	U6-gRNA	*3xP3-GFP*	*kynurenine hydroxylase^white^*	white-eye	G_1_: 99.5% G_3_: 98.8% G_4_: 97.2%	[34]
*Anopheles gambiae*	CRISPR^h^	*vasa2*-Cas9	U6-gRNA	*3xP3-RFP*	*AGAP007280* *AGAP005958* *AGAP011377*	sterility	G_2_: 99%, G_3_: 97.6% G_2_: 95.8%, G_3_: 92.8% G_2_: 82.8%, G_3_: 75.5%,	[35]
*Anopheles gambiae*	dsxF^CRISPRh^	*zpg*-Cas9	U6-gRNA	*3xP3-RFP*	*doublesex*	sterility in homozygous females	95.9% in ♂; 99.4% in ♀	[36]
*Anopheles gambiae*	SDGD^dsx^	*zpg*-Cas9	U6-gRNA	*3xP3-DsRed*	*doublesex* X chromosome	distort sex ratios (male only)	92% in ♂; 99% in ♀	[61]
*Anopheles gambiae*	AgNosCd-1	*nanos*-Cas9	U6-gRNA	*3xP3-CFP*	*Agcd*	red-eye	96.7% (G_1_–G_4_)	[48]
*Anopheles stephensi*	Re*ckh*	*vasa*-Cas9	U6A-gRNA	*3xP3-GFP*	*kynurenine hydroxylase^white^*	white-eye	99.8% in ♂; 57% in ♀	[49]
*Anopheles gambiae*	*nos*-CRISPR^h^ *zpg*-CRISPR^h^ *exu*-CRISPR^h^	*zpg*-Cas9 *nanos*-Cas9 exu-Cas9	U6-gRNA	*3xP3-DsRed*	*AGAP007280*	recessive female sterility	93.5% in ♂, 97.8% in ♀; 99.6% in ♂, 99.1% in ♀; 65.0% in ♂; 0 in ♀	[50]
*Ceratitis capitata*	–	*vasa-Cas9*	U6-gRNA	*pUb-DsRed*	*transformer*	sex conversion from females to males	83.1%	[40]
*Plutella xylostella*	–	*vasa-Cas9* *meiw68-Cas9* *nanos-Cas9*	U6-gRNA	*Hr5ie1-DsRed*	*yellow* *kmo*	yellow-pigmentation yellow-eye	no significant deviation from 50% inheritance	[41]
*Plutella xylostella*	–	*nanos-Cas9*	U6-gRNA	*Hr5ie1-EGFP*	*yellow*	yellow-pigmentation	6.67–12.59%	[51]

–: not available; ♀: female; ♂: male.

## Data Availability

All data were sourced from the scientific literature referenced in the References section. No additional data were generated.
